# The impact of ibezapolstat and other *Clostridioides difficile* infection-relevant antibiotics on the microbiome of humanized mice

**DOI:** 10.1128/aac.01604-24

**Published:** 2025-02-25

**Authors:** Trenton M. Wolfe, Jinhee Jo, Nick V. Pinkham, Kevin W. Garey, Seth T. Walk

**Affiliations:** 1 Department of Microbiology and Cell Biology, Montana State University 123776 , Bozeman, Montana, USA; 2 Department of Pharmacy Practice and Translational Research, University of Houston College of Pharmacy 15507 , Houston, Texas, USA; Columbia University Irving Medical Center, New York, New York, USA

**Keywords:** *Clostridioides difficile *infection, microbiome, ibezapolstat, fidaxomicin, germ-free mouse

## Abstract

Ibezapolstat (IBZ) is a competitive inhibitor of the bacterial Pol IIIC enzyme in clinical development for the treatment of *Clostridioides difficile* infection (CDI). Previous studies demonstrated that IBZ carries a favorable microbiome diversity profile compared to vancomycin (VAN). However, head-to-head comparisons with other CDI antibiotics have not been done. The purpose of this study was to compare microbiome changes associated with IBZ to other clinically used CDI antibiotics. Groups of germ-free (GF) mice received a fecal microbiota transplant from one of two healthy human donors and were subsequently exposed to either IBZ, VAN, fidaxomicin (FDX), metronidazole (MET), or no antibiotic (control). 16S rRNA encoding gene sequencing of temporally collected stool samples was used to compare the gut microbiome perturbations between treatment and no-drug control groups. Among the tested antibiotics, the most significant change in microbiome diversity was observed in MET-treated mice. Each antibiotic had a unique effect, but changes in alpha and beta diversities following FDX- and IBZ-treated groups were less pronounced than those observed in VAN- or MET-treated groups. By the end of therapy, both IBZ and FDZ increased the relative abundance of *Bacteroidota* (phylum), with IBZ additionally increasing the relative abundance of *Actinomycetota* (phylum). In microbiome-humanized mice, IBZ and FDX had smaller effects on gut microbiome diversity than VAN and MET. Notable differences were observed between the microbiome of IBZ- and FDX-treated groups, which may allow for differentiation of these two antibiotics in future studies.

## INTRODUCTION

*Clostridioides difficile* is a gram-positive, spore-forming, anaerobic bacterium and a leading cause of healthcare-associated and community infections (1). *C. difficile* infection (CDI) occurs when ingested spores germinate in the gut in response to changes in the gut microbiome induced primarily by antibiotics (2). Two major bacterial phyla, *Bacillota* and *Bacteroidota*, are typically the most negatively affected by common CDI-predisposing antibiotics, while the relative abundance of other and typically rare phyla, like *Pseudomonadota*, increases (3). CDI is often treated with antibiotics, including vancomycin (VAN) or fidaxomicin (FDX). Metronidazole (MET) was previously a treatment of choice; however, it is no longer recommended and only used in certain settings. This can be attributed to decreased susceptibility against *C. difficile* (4) and decreased levels of MET in the stool (5) (indicating its decreased efficacy for pathogens of the large intestine, such as *C. difficile*). The ideal antibiotic for CDI treatment would selectively target *C. difficile* while preserving the rest of the microbiome, thus allowing for recovery of microbiome diversity and preventing CDI recurrence. Thus, the development of new CDI antibiotics aims to minimize impacts on the microbiome. However, the distinct mechanisms of action of these drugs lead to varying effects on microbiome taxa. A direct way to evaluate a drug’s selectivity is to quantify changes in ecologic diversity (i.e., richness and evenness of microbiome taxa) between treated and untreated individuals, also known as beta diversity. FDX has been approved for the treatment of CDI in 2011 and is now the prototypical narrow-spectrum antibiotic for CDI. This macrolide targets bacterial RNA polymerase (RNAP), which in turn inhibits transcription. A conserved residue in RNAP confers greater specificity to *C. difficile* and other Bacillota as opposed to Bacteroidota (6). This contrasts with the broad-spectrum activity of VAN, a glycopeptide that binds lipid-II-D-Ala-D-Ala to halt cell wall formation. VAN is active against both Bacteroidota and Bacillota (7). MET, a nitroimidazole prodrug, is activated within cells by oxidoreductases to produce reactive species that cause DNA and protein damage, depletion of thiols, and eventually lead to cell death (8). MET has been shown to reduce *Bacillota* but not *Bacteroidota* or *Pseudomonadota* (9).

Ibezapolstat (IBZ) is a novel antibiotic currently in clinical development for the treatment of CDI (10). IBZ acts as a competitive inhibitor of the C-family DNA polymerase IIIC (Pol IIIC) DNA synthesis substrate 2′-deoxyguanosine 5′-triphospate (dGTP) (11). The IBZ base pairing domain mimics guanine, resulting in the formation of an inactive ternary complex of IBZ, DNA, and PolC. *pol IIIC* is an attractive antibiotic target, as it is present in the phylum Bacillota, which includes *C. difficile*, and absent in other phyla common to the human gut (*Actinomycetota*, *Bacteroidota*, and *Pseudomonadota*) (12). In clinical trials, IBZ demonstrated targeted activity within the *Bacillota* phylum (10, 13, 14), selectively killing *C. difficile* while maintaining or even increasing the relative abundance of closely related Pol IIIC *Bacillota* species known to be important in preventing recurrent CDI.

Despite the importance of microbiome disruption in the pathogenesis of CDI, a head-to-head comparison of all four CDI-relevant antibiotics on the same human microbiome has not been evaluated. Such a comparison is challenging in CDI patients due to inter-individual variability in microbiome composition and the diverse effects of CDI-predisposing antibiotics (i.e., before CDI onset). In this study, we treated two different groups of microbiome-humanized mice (i.e., germ-free recipients of human fecal transplants) with IBZ, FDX, VAN, MET, or untreated (no-drug) controls. While all antibiotics significantly impacted microbiome diversity, both IBZ and FDX resulted in significantly less perturbation than VAN or MET. These results are consistent with clinical trial results and demonstrate the value of humanized mice for the preclinical evaluation of microbiome perturbation when developing prototypical therapeutics.

## MATERIALS AND METHODS

### Animals

**Fig 1:**
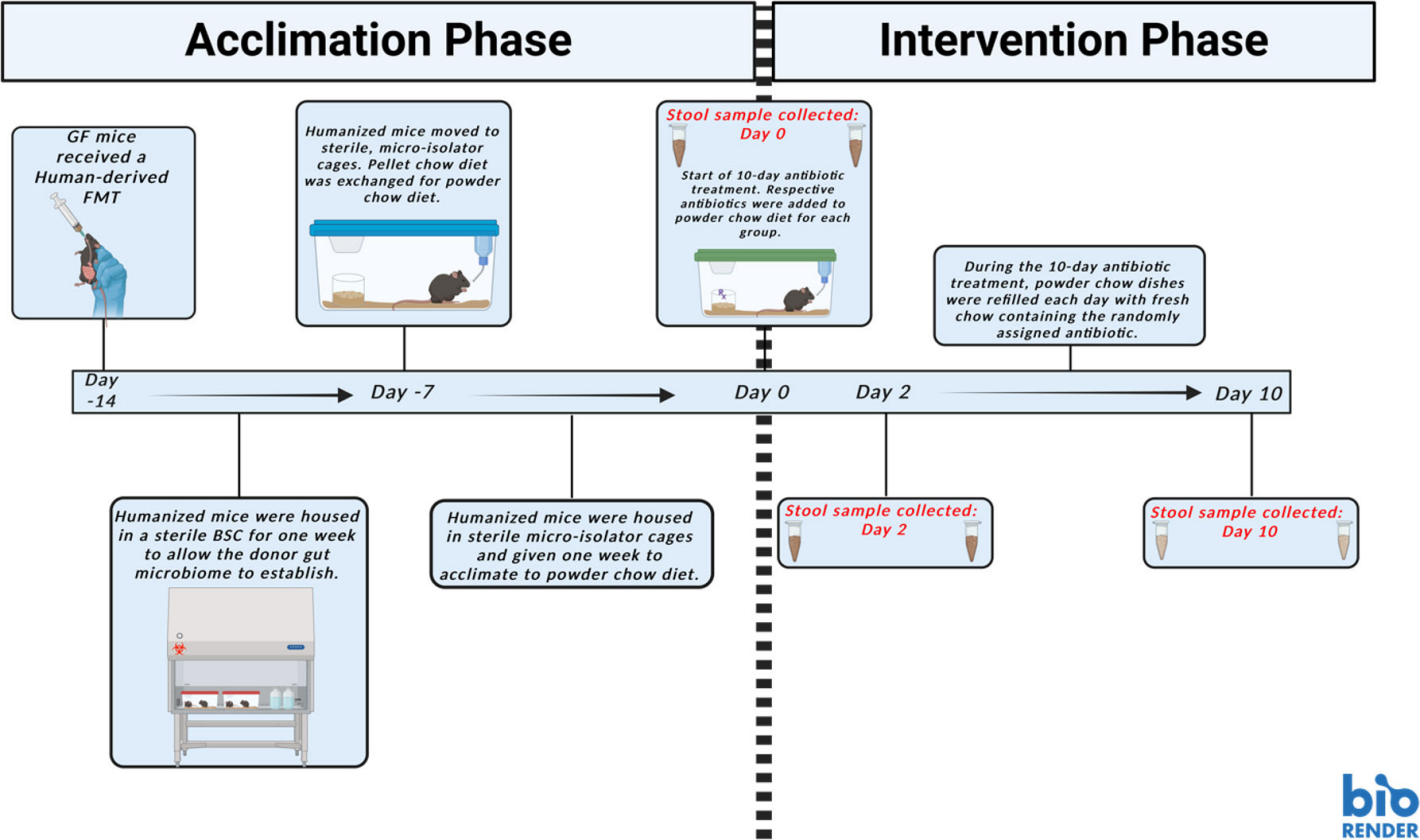
Graphical illustration of the humanized mouse model used in this study. Germ-free (GF) mice were engrafted with a healthy human donor microbiome via fecal microbiota transplant (FMT). Following FMT, mice were given 7 days to establish a baseline microbiome. From there, conventional pellet-style chow was exchanged for the powder style. Another 7 days were given for the natural and expected shift of the microbiome following this change in diet. At treatment day 0, a stool sample was collected to establish baseline microbiome. Mice were randomly assigned to an antibiotic-treatment group, and stool samples were collected at treatment days 2 and 10. This figure was created with BioRender.com.

Animal experiments were conducted at Montana State University’s Animal Resource Center, an American Association for the Accreditation of Laboratory Animal Care-accredited facility. GF mice were housed in hermetically sealed and HEPA-filter ventilated vinyl isolators (Park Bio, Groveland, MA) and fed sterile food (5010, Laboratory Autoclavable Rodent Diet, LabDiet, St. Louis, MO) and sterile water. All food and water supplied to GF mice were quarantined, monitored, and tested for microbial contamination via cultivation-dependent and -independent methods. For humanization, mice were given 100 µL of thawed human stool mixed at 1:1 g stool per volume of anaerobic PBS as previously described (15). Following humanization, mice were kept in a pre-sterilized biosafety cabinet. Due to the water insolubility of some of the antibiotics, clinically used antibiotic tablets were pulverized and mixed into a powdered mouse diet (AIN-93G Purified Rodent Diet, Dyets, Bethlehem, PA) and delivered daily in powder diet dishes (#910018 Complete Jar Set, Dyets, Bethlehem, PA). Based on a previous study (16), mice were acclimated twice, that is, once to humanization (7 days) and subsequently to the powdered diet (7 days) prior to antibiotic administration, to account for microbiome changes due to switch to powdered diet. Mice were housed under specific pathogen-free conditions (including murine norovirus) in individually ventilated, sterilized cages with food and water provided *ad libitum*. Mice were treated with antibiotics for 10 days, the guideline-recommended duration for CDI. A graphical illustration of the study design is provided in Fig. 1.

### Human samples and fecal microbiota transplantation (FMT)

Two frozen stool samples from healthy male adults (22 and 44 years of age) participating in a previous IRB-approved study (17) were used to humanize mice via FMT in two trials. Samples were initially processed inside an anaerobic chamber and stored at −80°C until use. The frozen stool was thawed inside an anaerobic chamber (Coy Laboratories), suspended in sterile, pre-reduced phosphate-buffered saline (PBS) at an approximate stool weight to a PBS volume ratio of 1:1, and briefly vortexed (<15 s) to generate a fecal slurry. 100 µL of this fecal slurry was used to inoculate 16 GF mice in each trial via oral gavage (Table S1).

### Pharmaceutical drugs and dosing

Human equivalent dosing of each drug was calculated based on US FDA “Guidance for Industry” (18). This allometric conversion adjusts drug doses in lab animals according to differences in body surface area between humans and mice. These recommendations were developed primarily for orally ingested drugs intended for systemic activity. We believe these recommendations are relevant for antibiotic administration because some orally ingested antibiotics are prescribed for systemic infections and because these drugs must reach the small intestine where they are absorbed. The same doses per gram body weight used to treat human CDI were converted to mouse equivalents (Table S2). Antibiotic tablets were ground via mortar and pestle into a fine powder, weighed, and mixed with the powder diet. Mice were randomized into five treatments groups, corresponding to four CDI antibiotics (IBZ, FDX, VAN, MET) or an untreated (control) group for 10 days per the Infectious Diseases Society of America–Society for Healthcare Epidemiology of America (IDSA-SHEA) CDI treatment guidelines (4). Fresh powdered diet of diet/antibiotic mixtures was supplied daily. Powdered pharmaceutical-grade antibiotics were added at the following concentrations: IBZ (45.34 mg/cage [*n* = 5]/day in 40 g powdered chow/day), FDX (20.15 mg/cage [*n* = 5]/day in 40 g powdered chow/day), VAN (25.19 mg/cage [*n* = 5]/day in 40 g powdered chow/day), and MET (50.37 mg/cage [*n* = 5]/day in 40 g powdered chow/day). An untreated control group served as a comparator (40 g of powdered chow/day). A more detailed table of drug dosing is provided in Table S2.

### 16S rRNA encoding gene sequencing

Unique ear punches identified individual mice, and stool samples were collected 7 days after acclimating to FMT, 7 days after acclimating to the powdered diet (day 0 of treatment), day 2 of antibiotic treatment, and day 10 of antibiotic treatment. The stool was obtained by humanely restraining mice and collecting pellets directly into sterile Eppendorf tubes. Samples were immediately frozen at –80°C until DNA extraction. Bulk DNA was extracted from thawed samples using the DNeasy PowerSoil Kit (Qiagen, Hilden, Germany). DNA was then frozen at –80°C and shipped overnight on dry ice to the University of Michigan Center for Microbial Systems for Illumina MiSeq amplicon sequencing of the V4 variable region of the 16S rRNA encoding gene (dual-indexed barcoded primers, 2 × 250 base-pair reads) (19). Raw sequencing reads were processed using mothur (v.1.48.0) (20) and the MiSeq Standard Operating Procedure (19) accessed July 11, 2023 (https://mothur.org/wiki/miseq_sop/). Paired-end reads were assembled into contigs, screened for length and quality (maximum 275 bp and minimum of 246 bp with no ambiguous base calls), and aligned to coordinates 1,968 through 11,550 of the Silva ribosomal RNA gene reference database (v.138.1). Potential chimeras were identified and removed using the Uchime algorithm via mothur (21). Non-target reads (i.e., mitochondria, chloroplast, *Eukaryota*, and sequences unclassified at the domain level) were removed, and OTUs were identified in mothur using the VSEARCH distance-based greedy clustering algorithm at the 97% sequence similarity threshold (22). From this, an OTU-based data matrix was built. Rare OTUs with <100 total reads were removed to minimize the influence of spurious observations. This resulted in the loss of one mouse in the IBZ-treated group in trial 2. Taxonomic classifications were assigned using the Bayesian classifier of the Ribosomal Database Project (23) implemented in mothur (training set v.18). Bray–Curtis distances between centroids were tabulated by first calculating the centroid of each group by averaging the abundance of each OTU across all communities in the group, and then taking the Bray–Curtis distance between the centroids.

### Statistical analysis

Microbiome analysis was done with R version 4.3.1. Diversity estimates were calculated with R’s vegan package (version 2.6–4). Alpha diversity was quantified with the inverse of the Simpson index and compared using *t*-tests. Beta diversity was quantified using Bray–Curtis dissimilarity, and PERMANOVA (using 999 permutations) was used to test for significance. Taxonomic changes were explored with Wilcox-ranked sum tests using the R package CAF’s (https://github.com/nvpinkham/CAF) taxonomy shared function. Statistical summaries are provided in the supplemental material.

## RESULTS

**Fig 2:**
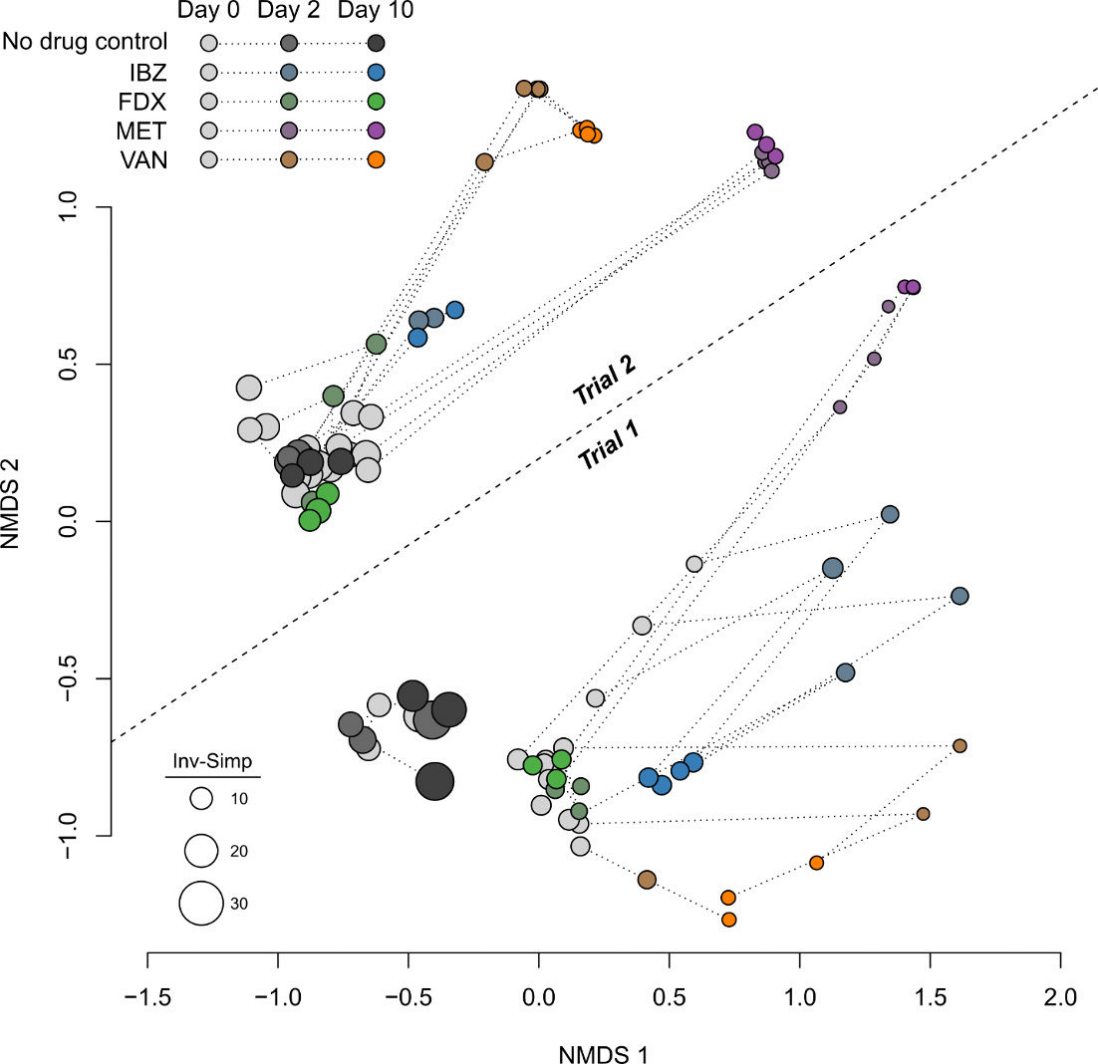
Non-metric multidimensional scaling (NMDS) of the beta-diversities between microbiome samples. Baseline (day 0) samples are colored gray for all treatment groups. Days 2 and 10 of treatments are colored as shown in the legend. Dotted lines connect samples from individual mice. Trials 1 and 2 microbiomes were distinct, as indicated by the labeled dashed line. The size of each dot was scaled according to alpha diversity (inverse Simpson’s index), with larger dots representing more diverse microbiomes.

**Fig 3:**
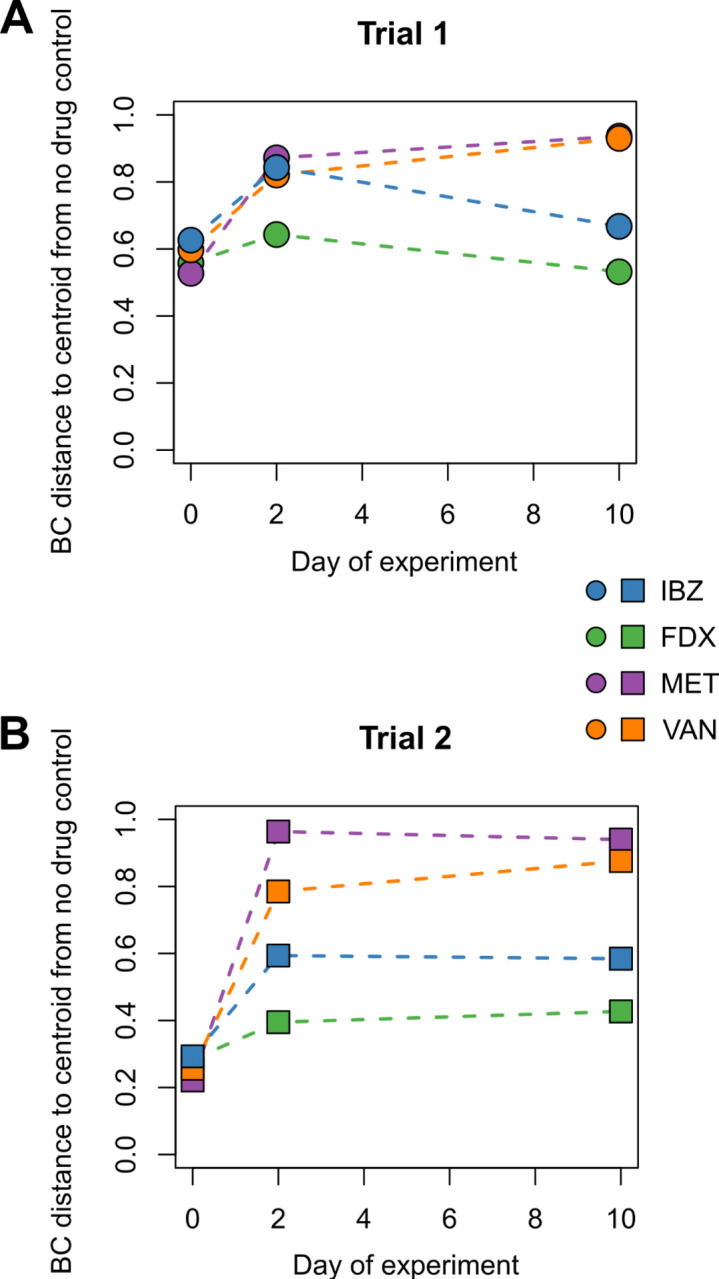
Bray–Curtis dissimilarity distance to treatment group centroids for each antibiotic relative to the no-drug control group. Trials 1 (**A**) and 2 (**B**) are shown separately.

**Fig 4:**
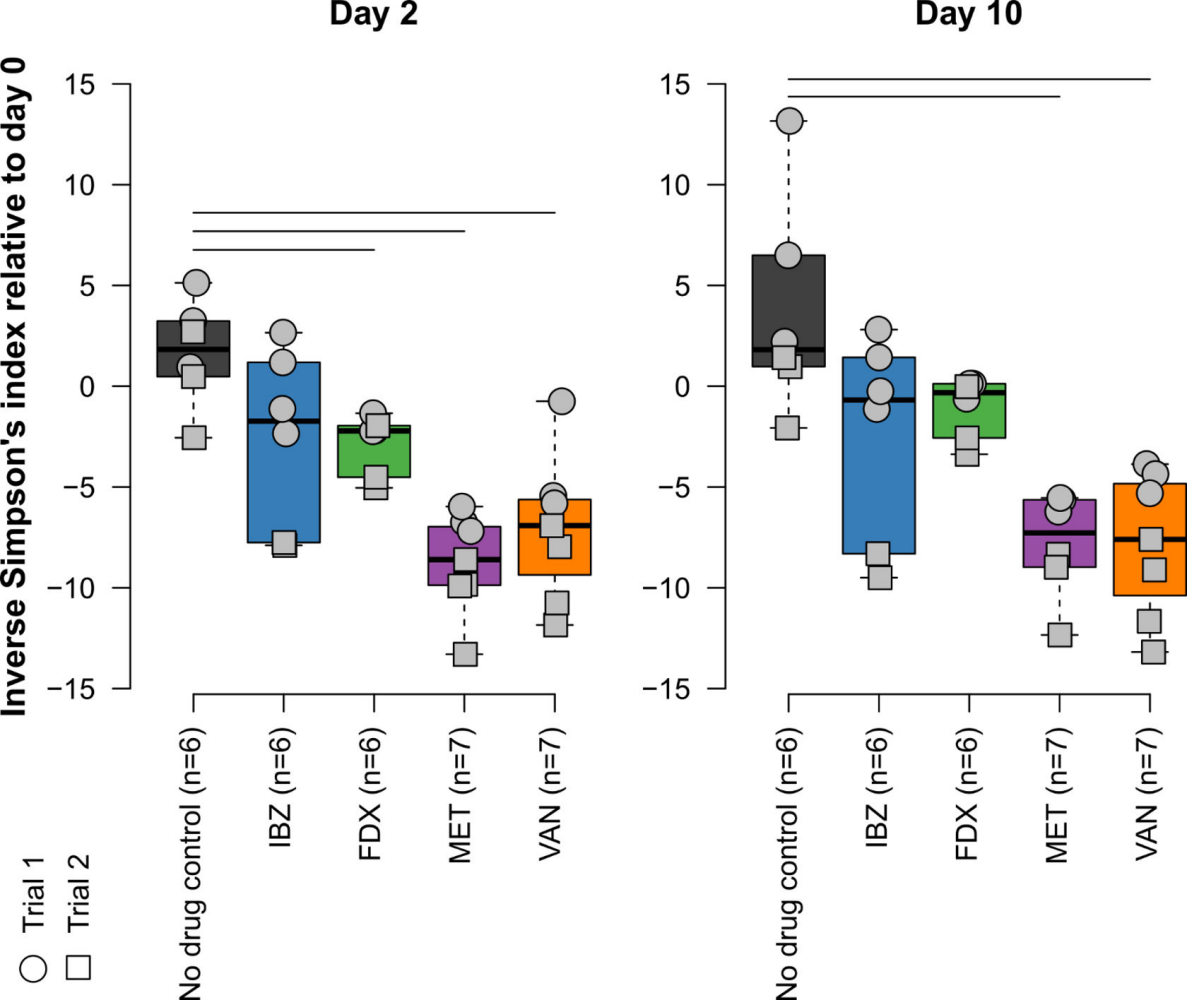
Alpha diversity (inverse Simpson’s index) for each treatment group on days 0 (top) and 10 (bottom) of treatment. Samples from individual mice were colored gray and shown according to trial (circle = trial 1, box = trial 2). Vertical lines represent statistically different (*P* < 0.05) group comparisons after adjustment for false discovery rate.

Two groups of GF mice (*n* = 16 each) were evaluated in separate experiments (trials 1 and 2). Each trial consisted of fecal microbiota transplant (FMT) from a healthy human donor, with a different donor sample used in each trial. All treatments were well tolerated, except for one instance. In trial 2, one mouse in the MET treatment group was found dead on day 3. A necropsy did not reveal any gross anatomical abnormalities, and the cause of death remained unclear. No other mice exhibited noticeable signs of disease. Additionally, one mouse from the IBZ treatment group in trial 2 was excluded from the analysis due to insufficient sequencing reads from stool samples. As expected, the microbiomes of humanized mice in trial 1 were significantly different from those in trial 2 before antibiotic treatment (Fig. 2, PERMANOVA: *R*^2^ = 0.592, *F*_tat_ = 43.598, *P* = 0.001), supporting that inter-individual differences in human donor microbiomes were successfully transferred to GF recipient mice via FMT. Cages of mice typically consumed all of the provided powdered chow, suggesting that optimal dosing was achieved when antibiotics were added. Antibiotic treatment effects were apparent by day 2 in both trials compared to untreated controls and persisted throughout the experiment until day 10 (Fig. 2). Bray–Curtis dissimilarity beta-diversity indicated that microbiomes from IBZ- and FDX-treated mice were more similar to the no-drug control group than VAN- and MET-treated mice (Fig. 3A and B). By the end of treatment, VAN- and MET-treated mice showed significantly higher Bray–Curtis distance from the no-drug control (*t*-test, *P* < 0.05), whereas IBZ- and FDX-treated mice did not show significant differences (Fig. 3A and B). Inverse Simpson’s index alpha diversity decreased in all antibiotic-treatment groups. However, IBZ did not show a significant difference from controls on day 2 or 10 of treatment (Fig. 4). Alpha diversity of the FDX-treated group was significantly different from controls on day 2 or 10, while VAN and MET were significantly different on both days (Fig. 4).

**Fig 5:**
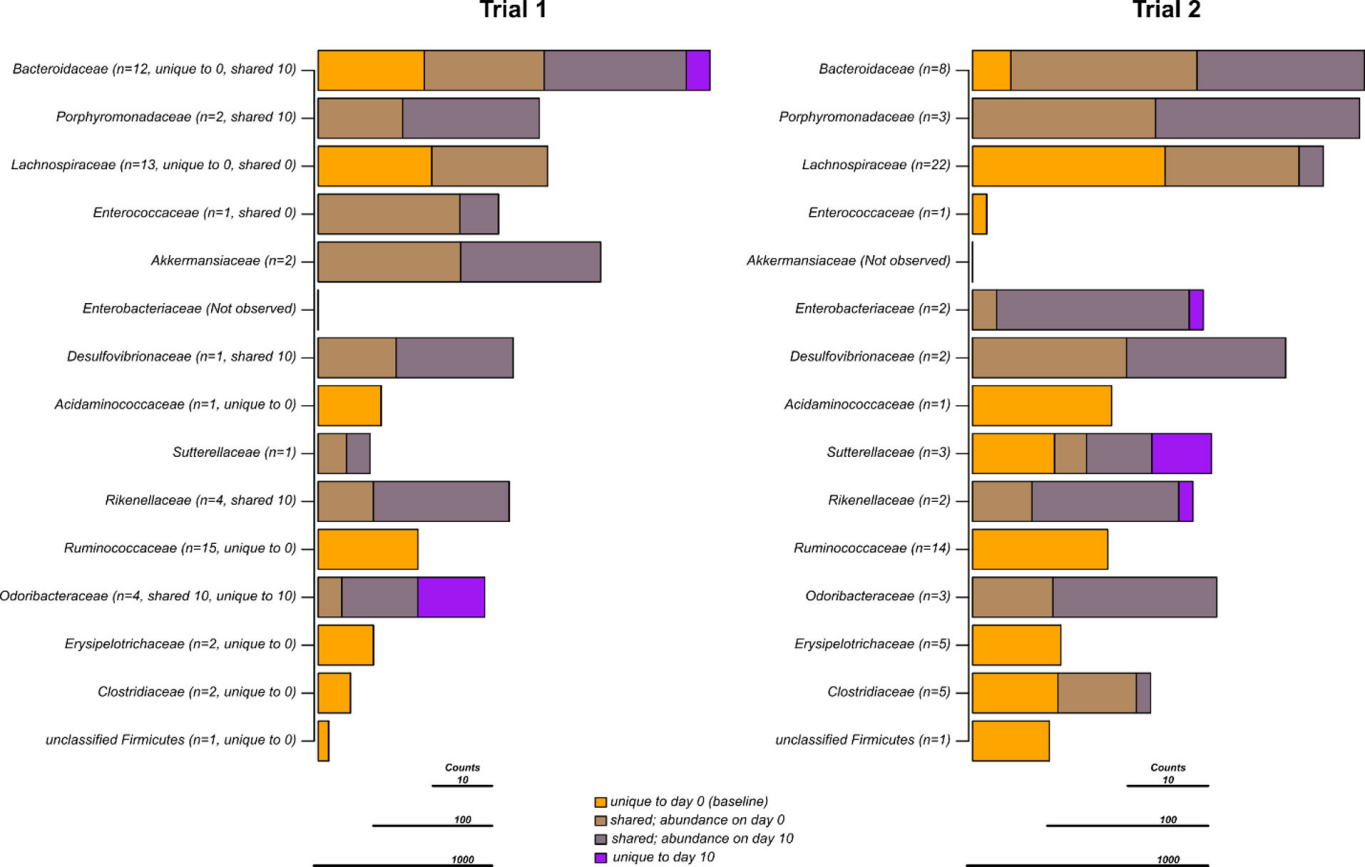
Unique and shared taxa on days 0 (baseline) and 10 (end of antibiotic exposure) for IBZ treatment. Each bar represents the median number of reads for the top 15 bacterial families observed in the study. For each family, stacked bars represent the median number of reads observed only on day 0 or 10 (i.e., unique) or observed on both days (i.e., shared). Shared bars are colored and scaled according to their relative abundance on either day 0 or 10, respectively. After each family name (in parentheses), the number of representative OTUs along with statistically significant (*P* < 0.05) results is shown (unique to 0 = significantly more OTUs observed on day 0 than on day 10, unique to 10 = significantly more OTUs observed on day 10 than on day 0, shared = an overabundance of reads on day 0 or 10 as indicated).

The presence–absence and relative abundance of the 15 most abundant families across both trials were evaluated (Fig. 5). In trial 1, IBZ treatment significantly affected all families, except *Akkermansiaceae* and *Sutterellaceae*. Additionally, seven of the 15 families exhibited significantly more reads unique to day 0, suggesting that IBZ reduced these taxa below the detection threshold. In contrast, *Odoribacteraceae* appeared to thrive with IBZ treatment, with significantly more reads unique to day 10. Similarly, shared taxa (observed on both days 0 and 10) from *Porphyromonadaceae*, *Desulfovibrionaceae*, *Rikenellaceae*, and *Odoribacteriaceae* were more abundant on day 10, suggesting they were unaffected by IBZ. Due to the limited number of mice (*n* = 2) treated with IBZ in trial 2, no statistical analysis was performed, though the trends largely mirrored those seen in trial 1, particularly for taxa unique to day 0.

## DISCUSSION

### FDX and IBZ exhibit lower microbiome disruption than VAN or MET

IBZ is a competitive inhibitor of the Pol IIIC DNA synthesis cognate substrate 2′-dGTP and has completed Phase 2 studies for the treatment of CDI in adults (11, 14). Results from the Phase I healthy volunteer study demonstrated distinct alpha and beta diversities between healthy volunteers given one of two doses of IBZ compared to those given oral VAN. This was characterized by increased abundance of *Actinomycetota* in IBZ-treated subjects and increased *Pseudomonadota* in VAN-treated subjects. In the Phase 2a study, CDI patients on IBZ therapy exhibited increased alpha diversity marked by the regrowth of beneficial *Bacillota* with an increase in secondary bile acid concentrations (10). Most clinical trial development in CDI includes VAN as a comparator, and the microbiome changes of IBZ compared to other CDI-directed antibiotics were not done during the clinical trial development process. To provide key insights into microbiome changes of IBZ compared to other CDI-directed antibiotics, including FDX, we used a humanized mouse model (GF mice receiving a human-derived FMT) to assess the microbiome changes associated with IBZ, FDX, MET, and VAN. Our results demonstrated that IBZ-treated mice showed alpha diversity changes comparable to FDX, with much less alteration than those treated with MET or VAN (Fig. 4). These differences in alpha diversity resulted in distinct beta diversity changes between treatment groups. Overall, IBZ and FDX had smaller beta diversity shifts from baseline than VAN or MET (Fig. 2 and 3). The taxonomic impacts of IBZ and FDX also differed, with varying regrowth patterns observed at the family level (Fig. 5). Our analysis places IBZ in a similar category of microbiome disruption as FDX, indicating a narrow spectrum of microbiome alteration compared to broader-spectrum agents like VAN and MET. This opens up opportunities for future studies to differentiate these antibiotics based on their distinct effects on gut microbial composition changes. In the Phase 2a trial, *Ruminococcaceae* (*Bacillota* phylum) was positively correlated with increased secondary bile acid concentrations, which has an inhibitory effect on *C. difficile* spore germination (14, 24).

### Humanized mouse models can discern antibiotic effects on the microbiome

In this study, we demonstrated complex and dynamic changes in the gut microbiome of GF mice following human-derived FMT in response to different antibiotic exposures. Mice in this study consumed all or nearly all of the powdered chow provided daily in dishes, suggesting that there was minimal experimental error due to the taste preference of the antibiotic formulations. The model was sensitive to microbiome changes over time, even in control arms, and also affected by dietary changes (i.e., pellet to powder style). This highlights the significant role of diet and laboratory conditions in shaping the trajectory of the microbiome (25, 26). Post-FMT microbiome compositions of recipient mice resembled that of the human donors primarily consisting of *Bacillota* and *Bacteroidota* phyla (27). However, differences were observed, such as the increased abundance of *Verrucomicrobiota* phylum in one trial, which emphasizes the need for sufficient sample sizes to account for baseline variability between experiments. Despite these variations, the mouse model produced results consistent with previous drug trials, allowing for direct comparisons between antibiotics that is nearly impossible to do with human subjects. For instance, our study found that mice treated with MET had the most drastic changes to their microbiome composition, which is similar to other studies (9). Others have shown that MET reduces *Bacillota* but not *Bacteroidota* or *Pseudomonadota* (9). Here, we showed that mice had higher levels of *Bacillota* at the end of treatment (Fig. 5). This conflicting result might be due to differences in baseline microbiomes between the mice used here and in other studies or could be due to differences in drug availability in the lower GI tract. In asymptomatic carriers of *C. difficile*, administration of oral MET did not inhibit colonization, and 90% of participants had no detectable MET in the stool (5). Similarly, in patients with CDI, low levels of MET were detected in stool during acute CDI but not at the end of treatment (28, 29). It was suggested that diarrhea and inflammation might contribute to the increased MET levels during acute infection, and some suggest that MET can be inactivated by some members of the gut microbiome (30). Oral administration of C^14^-labeled MET to rats suggests that following rapid absorption, MET is secreted unchanged throughout the GI and vaginal tracts (31). Taken together, it seems possible that while MET levels are not high in stool, they may still be antimicrobial when arriving back in the gut following circulation.

### Study limitations

Our study has several important limitations. We chose to humanize mice with healthy human microbiomes to simulate a Phase-1 healthy-volunteer trial. Future experiments are needed to investigate drug efficacy (e.g., cure rate) or whether antibiotic-perturbed microbiomes influence *C. difficile* colonization and pathogenesis. We observed microbiome changes consistent with published human data on IBZ and other anti-CDI agents, but additional experiments are needed to test how consistent this is across human donors. Ideally, these analyses would also include metabolomic and other functional indicators of gut/microbiome health.

Using a humanized mouse model, we demonstrated that IBZ has a similarly narrow spectrum of activity to FDX as opposed to the broad-spectrum effect of VAN and MET. Significant differences were observed between the metagenomic data for IBZ and FDX, which may allow for further differentiation of these two narrow-spectrum antibiotics in future studies. This study also highlights the utility of humanized mouse models for evaluating the impact of antibiotics on the gut microbiome, closely mimicking the known effects in humans.

## Data Availability

All 16S rDNA sequencing reads were deposited in the National Center for Biotechnology Information (NCBI) BioProject database with accession code PRJNA934954. The authors declare that all other data supporting the study’s findings are available within this publication and its supplemental material or from the corresponding authors upon request.

## SUPPLEMENTAL MATERIAL

Supplemental material aac.01604-24-s0001.docx Figures S1 to S6; Tables S1 to S5.

ASM does not own the copyrights to Supplemental Material that may be linked to, or accessed through, an article. The authors have granted ASM a non-exclusive, world-wide license to publish the Supplemental Material files. Please contact the corresponding author directly for reuse.

## References

[1] Guh AY, Mu Y, Winston LG, Johnston H, Olson D, Farley MM, Wilson LE, Holzbauer SM, Phipps EC, Dumyati GK, Beldavs ZG, Kainer MA, Karlsson M, Gerding DN, McDonald LC, Emerging Infections Program Clostridioides difficile Infection Working Group. 2020. Trends in U.S. burden of Clostridioides difficile infection and outcomes. N Engl J Med 382:1320–1330. doi:10.1056/NEJMoa1910215 32242357 PMC7861882

[2] Britton RA, Young VB. 2014. Role of the intestinal microbiota in resistance to colonization by Clostridium difficile. Gastroenterology 146:1547–1553. doi:10.1053/j.gastro.2014.01.059 24503131 PMC3995857

[3] Panda A, Islam ST, Sharma G. 2022. Harmonizing prokaryotic nomenclature: fixing the fuss over phylum name flipping. MBio 13:e0097022. doi:10.1128/mbio.00970-22 35536003 PMC9239268

[4] Johnson S, Lavergne V, Skinner AM, Gonzales-Luna AJ, Garey KW, Kelly CP, Wilcox MH. 2021. Clinical practice guideline by the Infectious Diseases Society of America (IDSA) and Society for Healthcare Epidemiology of America (SHEA): 2021 focused update guidelines on management of Clostridioides difficile infection in adults. Clin Infect Dis 73:e1029–e1044. doi:10.1093/cid/ciab549 34164674

[5] Johnson S, Homann SR, Bettin KM, Quick JN, Clabots CR, Peterson LR, Gerding DN. 1992. Treatment of asymptomatic Clostridium difficile carriers (fecal excretors) with vancomycin or metronidazole. A randomized, placebo-controlled trial. Ann Intern Med 117:297–302. doi:10.7326/0003-4819-117-4-297 1322075

[6] Cao X, Boyaci H, Chen J, Bao Y, Landick R, Campbell EA. 2022. Basis of narrow-spectrum activity of fidaxomicin on Clostridioides difficile. Nature New Biol 604:541–545. doi:10.1038/s41586-022-04545-z PMC9635844 35388215

[7] Isaac S, Scher JU, Djukovic A, Jiménez N, Littman DR, Abramson SB, Pamer EG, Ubeda C. 2017. Short- and long-term effects of oral vancomycin on the human intestinal microbiota. J Antimicrob Chemother 72:128–136. doi:10.1093/jac/dkw383 27707993 PMC5161046

[8] Olaitan AO, Dureja C, Youngblom MA, Topf MA, Shen WJ, Gonzales-Luna AJ, Deshpande A, Hevener KE, Freeman J, Wilcox MH, Palmer KL, Garey KW, Pepperell CS, Hurdle JG. 2023. Decoding a cryptic mechanism of metronidazole resistance among globally disseminated fluoroquinolone-resistant Clostridioides difficile. Nat Commun 14:4130. doi:10.1038/s41467-023-39429-x 37438331 PMC10338468

[9] Huang C, Feng S, Huo F, Liu H. 2022. Effects of four antibiotics on the diversity of the intestinal microbiota. Microbiol Spectr 10:e0190421. doi:10.1128/spectrum.01904-21 35311555 PMC9045271

[10] Garey KW, McPherson J, Dinh AQ, Hu C, Jo J, Wang W, Lancaster CK, Gonzales-Luna AJ, Loveall C, Begum K, Jahangir Alam M, Silverman MH, Hanson BM. 2022. Efficacy, safety, pharmacokinetics, and microbiome changes of ibezapolstat in adults with Clostridioides difficile infection: a phase 2a multicenter clinical trial. Clin Infect Dis 75:1164–1170. doi:10.1093/cid/ciac096 35134880 PMC9525077

[11] Torti A, Lossani A, Savi L, Focher F, Wright GE, Brown NC, Xu WC. 2011. Clostridium difficile DNA polymerase IIIC: basis for activity of antibacterial compounds. Curr Enzym Inhib 7:147–153. doi:10.2174/157340811798807597 22844265 PMC3404731

[12] Timinskas K, Balvočiūtė M, Timinskas A, Venclovas Č. 2014. Comprehensive analysis of DNA polymerase III α subunits and their homologs in bacterial genomes. Nucleic Acids Res 42:1393–1413. doi:10.1093/nar/gkt900 24106089 PMC3919608

[13] Garey KW, Begum K, Lancaster C, Gonzales-Luna A, Bui D, Mercier J, Seng Yue C, Ducharme MP, Hu M, Vince B, Silverman MH, Alam MJ, Kankam M. 2020. A randomized, double-blind, placebo-controlled, single and multiple ascending dose Phase 1 study to determine the safety, pharmacokinetics and food and faecal microbiome effects of ibezapolstat administered orally to healthy subjects. J Antimicrob Chemother 75:3635–3643. doi:10.1093/jac/dkaa364 32892222 PMC7662179

[14] McPherson J, Hu C, Begum K, Wang W, Lancaster C, Gonzales-Luna AJ, Loveall C, Silverman MH, Alam MJ, Garey KW. 2022. functional and metagenomic evaluation of ibezapolstat for early evaluation of anti-recurrence effects in Clostridioides difficile infection. Antimicrob Agents Chemother 66:e0224421. doi:10.1128/aac.02244-21 35862742 PMC9380534

[15] Coryell M, McAlpine M, Pinkham NV, McDermott TR, Walk ST. 2018. The gut microbiome is required for full protection against acute arsenic toxicity in mouse models. Nat Commun 9:5424. doi:10.1038/s41467-018-07803-9 30575732 PMC6303300

[16] Yan L, Combs GF, DeMars LC, Johnson LK. 2011. Effects of the physical form of the diet on food intake, growth, and body composition changes in mice. J Am Assoc Lab Anim Sci 50:488–494. 21838977 PMC3148648

[17] Martinson JNV, Walk ST. 2020. Escherichia coli residency in the gut of healthy human adults. EcoSal Plus 9. doi:10.1128/ecosalplus.ESP-0003-2020 PMC7523338 32978935

[18] US Food and Drug Administration. 2005. Guidance for industry: estimating the maximum safe starting dose in initial clinical trials for therapeutics in adult healthy volunteers. U.S. Dept. of Health and Human Services, Food and Drug Administration, Center for Drug Evaluation and Research, Rockville, MD.

[19] Kozich JJ, Westcott SL, Baxter NT, Highlander SK, Schloss PD. 2013. Development of a dual-index sequencing strategy and curation pipeline for analyzing amplicon sequence data on the MiSeq Illumina sequencing platform. Appl Environ Microbiol 79:5112–5120. doi:10.1128/AEM.01043-13 23793624 PMC3753973

[20] Schloss PD, Westcott SL, Ryabin T, Hall JR, Hartmann M, Hollister EB, Lesniewski RA, Oakley BB, Parks DH, Robinson CJ, Sahl JW, Stres B, Thallinger GG, Van Horn DJ, Weber CF. 2009. Introducing mothur: open-source, platform-independent, community-supported software for describing and comparing microbial communities. Appl Environ Microbiol 75:7537–7541. doi:10.1128/AEM.01541-09 19801464 PMC2786419

[21] Edgar RC, Haas BJ, Clemente JC, Quince C, Knight R. 2011. UCHIME improves sensitivity and speed of chimera detection. Bioinformatics 27:2194–2200. doi:10.1093/bioinformatics/btr381 21700674 PMC3150044

[22] Westcott SL, Schloss PD. 2015. De novo clustering methods outperform reference-based methods for assigning 16S rRNA gene sequences to operational taxonomic units. PeerJ 3:e1487. doi:10.7717/peerj.1487 26664811 PMC4675110

[23] Wang Q, Garrity GM, Tiedje JM, Cole JR. 2007. Naive Bayesian classifier for rapid assignment of rRNA sequences into the new bacterial taxonomy. Appl Environ Microbiol 73:5261–5267. doi:10.1128/AEM.00062-07 17586664 PMC1950982

[24] Winston JA, Theriot CM. 2016. Impact of microbial derived secondary bile acids on colonization resistance against Clostridium difficile in the gastrointestinal tract. Anaerobe 41:44–50. doi:10.1016/j.anaerobe.2016.05.003 27163871 PMC5050083

[25] Ianiro G, Punčochář M, Karcher N, Porcari S, Armanini F, Asnicar F, Beghini F, Blanco-Míguez A, Cumbo F, Manghi P, Pinto F, Masucci L, Quaranta G, De Giorgi S, Sciumè GD, Bibbò S, Del Chierico F, Putignani L, Sanguinetti M, Gasbarrini A, Valles-Colomer M, Cammarota G, Segata N. 2022. Variability of strain engraftment and predictability of microbiome composition after fecal microbiota transplantation across different diseases. Nat Med 28:1913–1923. doi:10.1038/s41591-022-01964-3 36109637 PMC9499858

[26] Singh RK, Chang HW, Yan D, Lee KM, Ucmak D, Wong K, Abrouk M, Farahnik B, Nakamura M, Zhu TH, Bhutani T, Liao W. 2017. Influence of diet on the gut microbiome and implications for human health. J Transl Med 15:73. doi:10.1186/s12967-017-1175-y 28388917 PMC5385025

[27] Rinninella E, Raoul P, Cintoni M, Franceschi F, Miggiano GAD, Gasbarrini A, Mele MC. 2019. What is the healthy gut microbiota composition? A changing ecosystem across age, environment, diet, and diseases. Microorganisms 7:14. doi:10.3390/microorganisms7010014 30634578 PMC6351938

[28] Bolton RP, Culshaw MA. 1986. Faecal metronidazole concentrations during oral and intravenous therapy for antibiotic associated colitis due to Clostridium difficile. Gut 27:1169–1172. doi:10.1136/gut.27.10.1169 3781329 PMC1433873

[29] Abujamel T, Cadnum JL, Jury LA, Sunkesula VCK, Kundrapu S, Jump RL, Stintzi AC, Donskey CJ. 2013. Defining the vulnerable period for re-establishment of Clostridium difficile colonization after treatment of C. difficile infection with oral vancomycin or metronidazole. PLoS ONE 8:e76269. doi:10.1371/journal.pone.0076269 24098459 PMC3788714

[30] Freeman J, Baines SD, Saxton K, Wilcox MH. 2007. Effect of metronidazole on growth and toxin production by epidemic Clostridium difficile PCR ribotypes 001 and 027 in a human gut model. J Antimicrob Chemother 60:83–91. doi:10.1093/jac/dkm113 17483547

[31] Ings RM, McFadzean JA, Ormerod WE. 1975. The fate of metronidazole and tis implications in chemotherapy. Xenobiotica 5:223–235. doi:10.3109/00498257509052069 1154802

