# Supplementary material for: The impact of ibezapolstat and other Clostridioides difficile infection-relevant antibiotics on the microbiome of humanized mice
Source: Antimicrob Agents Chemother. 2025 Feb 25;69(4):e01604-24. doi: 10.1128/aac.01604-24 (PMC11963539; doi:10.1128/aac.01604-24)
Supplement: Supplemental material — Figures S1 to S6; Tables S1 to S5. [file aac.01604-24-s0001.docx]

**Supplemental Table 1.** Breakdown of mice used in each trial and randomly assigned treatment group.

| **Treatment Group** | **Trial 1** | **Trial 2** | **Grand Total** |
| --- | --- | --- | --- |
| Metronidazole | 3 Males | 4* Females | 6 |
| Fidaxomicin | 3 Females | 3 Males | 6 |
| No Drug Control | 3 Females | 3 Males | 6 |
| Ibezapolstat | 4 Males | 2* Females | 6 |
| Vancomycin | 3 Females | 4 Males | 7 |
| **Grand Total** | **16** | **16*** | **32*** |

*^*One mouse in the MTZ group of Trial 2 was found dead on day 3 of antibiotic treatment. An autopsy was performed, but no abnormal findings were found. For day 10 of the analysis, no sample was collected for this mouse, and tissues for histology were not collected for it. An insufficient read count was found for one of the mice in the IBZ-treated group in trial 2. Because of this, only n=2 is reported here as that was the number considered for microbiome analysis.^*

**Supplemental Table 2** Human Equivalent Dosing for Mice

**A. Human Equivalent Dosing (HED) conversion of human to mouse**


**B. Calculations for HED in powdered chow**

**
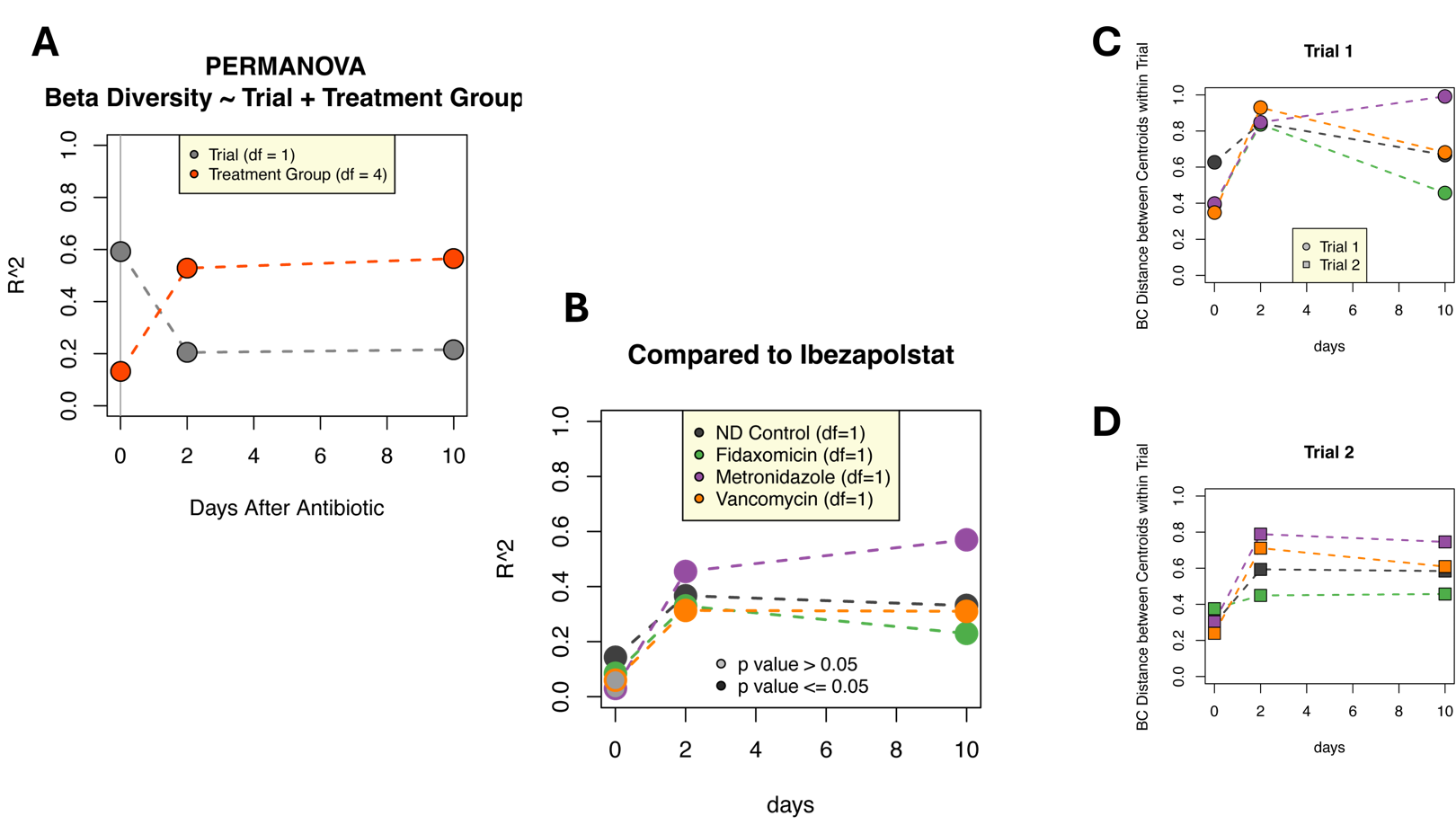
Supplemental Figure 1** Supplemental Data for Figure 1

**A.** R^2^ value for the PERMANOVA model BC ~ Trial + Treatment group results

of the trial group role in explaining beta diversity (Grey) and the treatment Group/Antibiotics (Red). Before antibiotic treatment, the majority of the variance was explained by differences between Trials 1 and 2. However, once antibiotic treatment started, the variance was primarily explained by antibiotic treatment over differences between trials.

**B.** R^2^ value comparing each treatment group to the IBZ treatment group.

**C.** Bray-Curtis Distance between centroids of each treatment group compared to the IBZ group in Trial 1. These are the same as Fig2AB of Fig1BC

**D.** Bray-Curtis Distance between centroids of each treatment group compared to the IBZ group in Trial 2.

**Supplemental Figure 2** Expanded Unique/Shared Analysis for the ND Control Groups


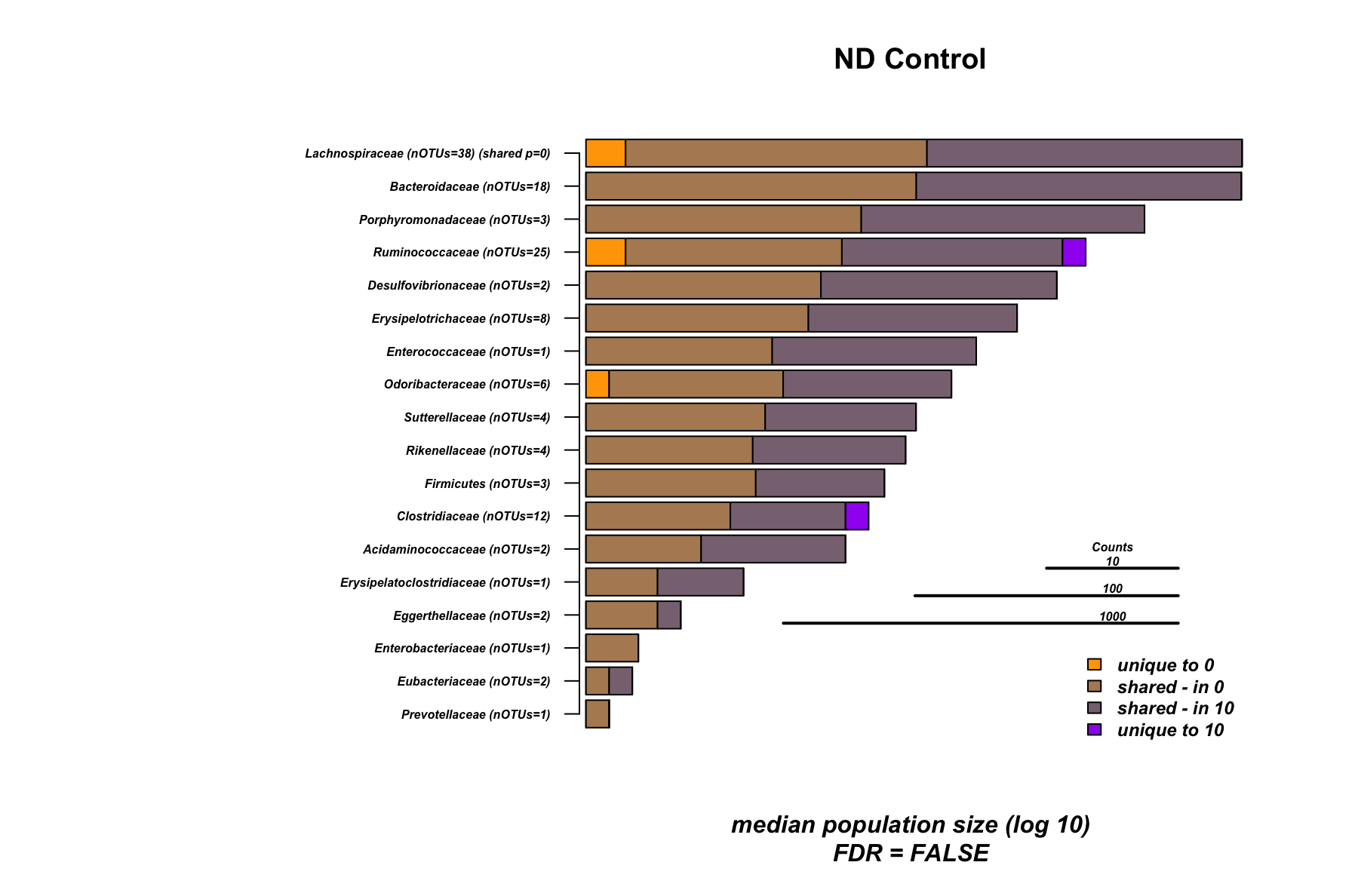


**Supplemental Figure 3** Expanded Unique/Shared Analysis for the Ibezapolstat Groups

- trials combined


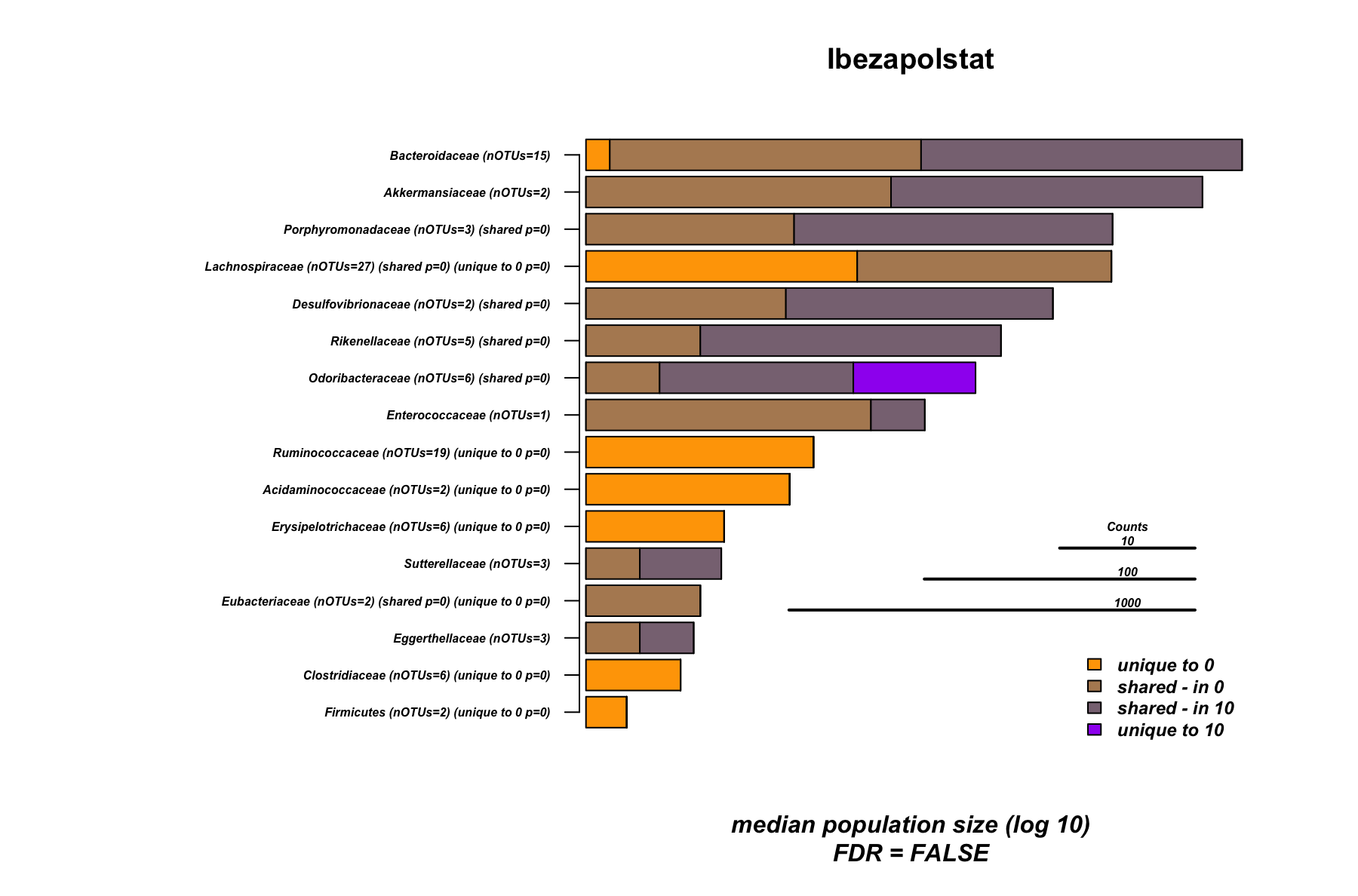


**Supplemental Figure 4** Expanded Unique/Shared Analysis for the Fidaxomicin Groups

**
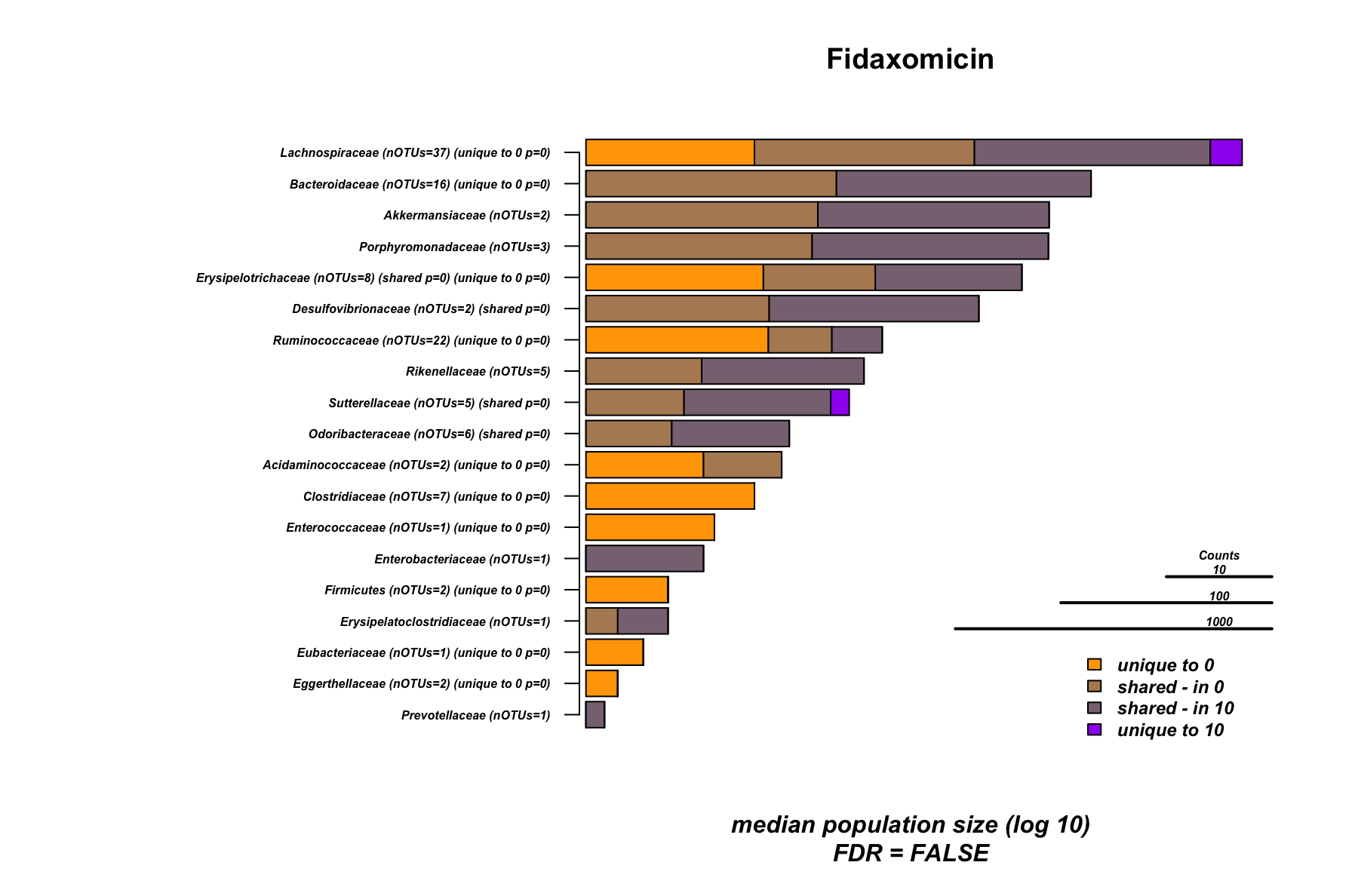
**

**Supplemental Figure 5** Expanded Unique/Shared Analysis for the Metronidazole Groups


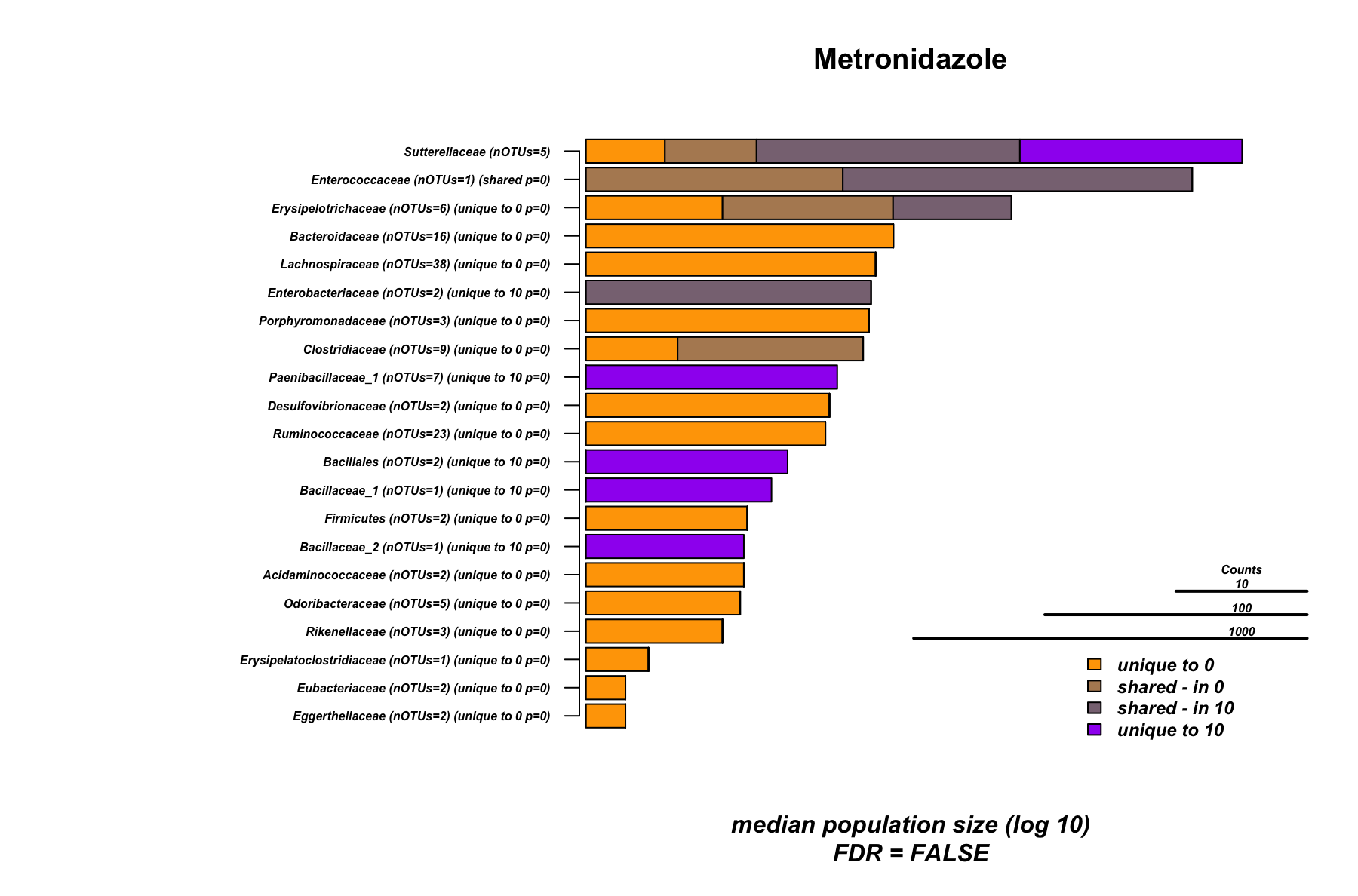


**Supplemental Figure 6** Expanded Unique/Shared Analysis for the Vancomycin Groups

**
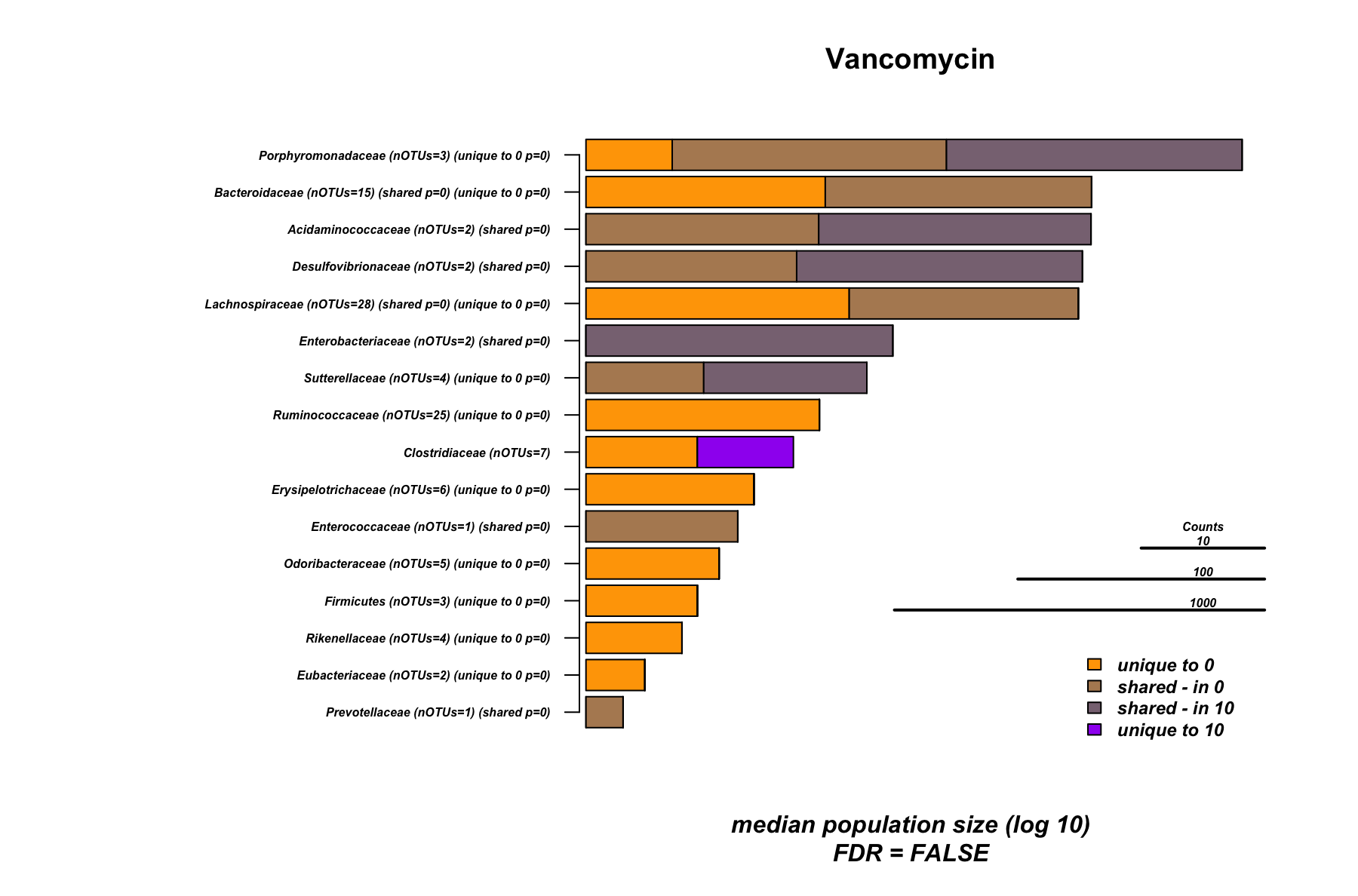
**

**Supplemental Table 3** Statistical Summary for Figure 1A (NMDS)

PERMANOVA results for Beta diversity ~ Trail + Antibiotic treatment

(Trial: DF= 1, R^2 = 0.22923, p = 0.001 ***) (Antibiotic treatment DF = 4, R2 = 0.25844, p = 0.001 ***)

Ordination stress plot:


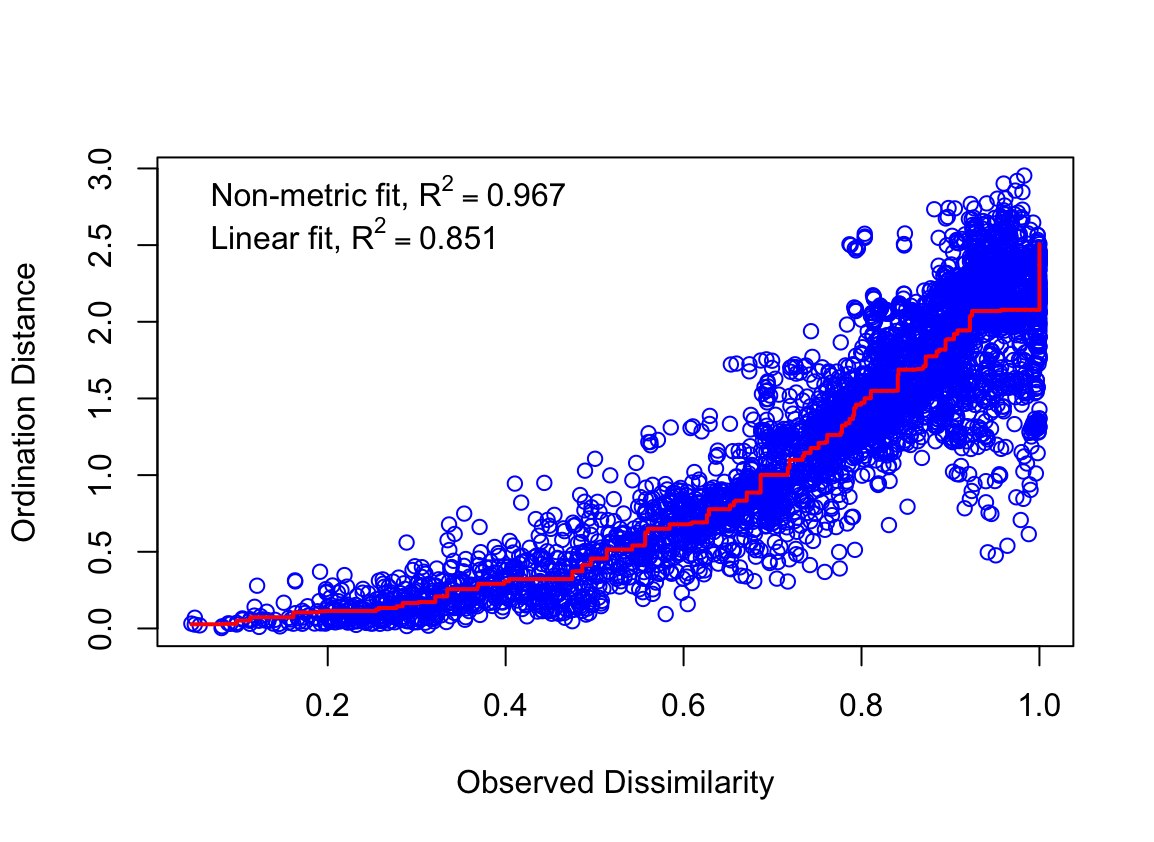


**Supplemental Table 4** T test Results for Figure 1D (Invsimp of Trial 1)

| **group1** | **group2** | **p** | **effect_size** | **t_stat** | **df** | **95_lo** | **95_hi** | **p.fdr** | **symbol** | **day** |
| --- | --- | --- | --- | --- | --- | --- | --- | --- | --- | --- |
| Metronidazole | Fidaxomicin | 0.0054 | 3.8563 | -7.2716 | 2.9926 | -5.5464 | -2.1663 | 0.0487 | * | 2 |
| Metronidazole | Ibezapolstat | 0.0041 | 4.8939 | -6.4175 | 3.6464 | -7.0946 | -2.6931 | 0.0414 | * | 2 |
| Metronidazole | Fidaxomicin | 0 | 4.8219 | -32.2418 | 3.9615 | -5.2387 | -4.4051 | 1E-04 | *** | 10 |
| Metronidazole | Ibezapolstat | 0 | 4.9426 | -33.548 | 3.9315 | -5.3545 | -4.5307 | 1E-04 | *** | 10 |
| Vancomycin | Fidaxomicin | 0 | 4.5601 | -25.9841 | 3.5737 | -5.0712 | -4.049 | 3E-04 | *** | 10 |
| Vancomycin | Ibezapolstat | 0 | 4.6808 | -26.9626 | 3.5072 | -5.1908 | -4.1709 | 3E-04 | *** | 10 |

**Supplemental Table 5** Statistical Summary for Figure 1E (Invsimp of Trial 2)

| **group1** | **group2** | **p** | **effect_size** | **t_stat** | **df** | **95_lo** | **95_hi** | **p.fdr** | **symbol** | **day** |
| --- | --- | --- | --- | --- | --- | --- | --- | --- | --- | --- |
| Metronidazole | Fidaxomicin | 0.0051 | 5.9191 | -9.0805 | 2.5723 | -8.2031 | -3.6352 | 0.0355 | * | 2 |
| Metronidazole | Ibezapolstat | 1E-04 | 4.2093 | -15.2429 | 3.9277 | -4.9817 | -3.437 | 0.0012 | ** | 2 |
| Vancomycin | Ibezapolstat | 8E-04 | 2.6291 | -9.1356 | 3.9751 | -3.4301 | -1.8281 | 0.0074 | ** | 2 |
| Vancomycin | Metronidazole | 0.0033 | -1.5802 | 4.7219 | 5.9795 | 0.7607 | 2.3998 | 0.0263 | * | 2 |
